# Probiotics for the Treatment of Atopic Dermatitis in Children: A Systematic Review and Meta-Analysis of Randomized Controlled Trials

**DOI:** 10.3389/fcimb.2017.00392

**Published:** 2017-09-06

**Authors:** Ruixue Huang, Huacheng Ning, Minxue Shen, Jie Li, Jianglin Zhang, Xiang Chen

**Affiliations:** ^1^Department of Occupational and Environmental Health, Xiangya School of Public Health, Central South University Changsha, China; ^2^Department of Dermatology, Xiangya Hospital, Central South University Changsha, China; ^3^Hunan Key Laboratory of Skin Cancer and Psoriasis, Xiangya Hospital, Central South University Changsha, China

**Keywords:** probiotics, constipation, children, meta-analysis, randomized controlled trial

## Abstract

**Objective:** Atopic dermatitis (AD) is a prevalent, burdensome, and psychologically important pediatric concern. Probiotics have been suggested as a treatment for AD. Some reports have explored this topic; however, the utility of probiotics for AD remains to be firmly established.

**Methods:** To assess the effects of probiotics on AD in children, the PubMed/Medline, Cochrane Library Scopus, and OVID databases were searched for reports published in the English language.

**Results:** Thirteen studies were identified. Significantly higher SCORAD values favoring probiotics over controls were observed (mean difference [MD], −3.07; 95% confidence interval [CI], −6.12 to −0.03; *P* < 0.001). The reported efficacy of probiotics in children < 1 year old was −1.03 (95%CI, −7.05 to 4.99) and that in children 1–18 years old was −4.50 (95%CI, −7.45 to −1.54; *P* < 0.001). Subgroup analyses showed that in Europe, SCORAD revealed no effect of probiotics, whereas significantly lower SCORAD values were reported in Asia (MD, −5.39; 95%CI, −8.91 to −1.87). *Lactobacillus rhamnosus* GG (MD, 3.29; 95%CI, −0.30 to 6.88; *P* = 0.07) and *Lactobacillus plantarum* (MD, −0.70; 95%CI, −2.30 to 0.90; *P* = 0.39) showed no significant effect on SCORAD values in children with AD. However, *Lactobacillus fermentum* (MD, −11.42; 95%CI, −13.81 to −9.04), *Lactobacillus salivarius* (MD, −7.21; 95%CI, −9.63 to −4.78), and a mixture of different strains (MD, −3.52; 95%CI, −5.61 to −1.44) showed significant effects on SCORAD values in children with AD.

**Conclusions:** Our meta-analysis indicated that the research to date has not robustly shown that probiotics are beneficial for children with AD. However, caution is needed when generalizing our results, as the populations evaluated were heterogeneous. Randomized controlled trials with larger samples and greater power are necessary to identify the species, dose, and treatment duration of probiotics that are most efficacious for treating AD in children.

## Introduction

Atopic dermatitis (AD), is one of the most common chronic inflammatory skin disorders among infants and children. AD is characterized by itching and recurrent eczematous lesions, and its incidence has increased worldwide over the past several decades. The current prevalence rate is 10–20% in infants and children (Weidinger and Novak, 2016). As the leading non-fatal medical skin disorder, AD imposes severe psychosocial burdens on pediatric patients and their families (Chamlin and Chren, 2010; Silverberg, 2016; Sidbury and Khorsand, 2017). AD is associated with high risks of allergy, asthma, and mental health issues (Sung et al., 2017). Infants and children with AD are typically treated with topical corticosteroids (TCS), antihistamines, and even antibiotics (Totri et al., 2017). However, these medications exert several adverse side effects, and AD symptoms may recur rapidly after treatment is stopped. Furthermore, long-term TCS use may trigger new-onset AD.

Probiotics is becoming increasingly attractive as a treatment option for some illnesses in children (Fuchs-Tarlovsky et al., 2016). Probiotics (live bacteria or yeasts) are not necessarily harmless, but they help to protect hosts from harmful bacteria (Mizock, 2015). When administered in adequate amounts, probiotics may play beneficial roles not only in the gastrointestinal tract but also in the gut–brain–skin axis (Ogden and Bielory, 2005; Dehingia et al., 2015; Huang et al., 2016; Huang and Hu, 2017). Several studies on the benefits of probiotics for pediatric AD patients have appeared over the past decades. In 2000, Pessi et al. reported that oral probiotics alleviated the clinical symptoms of gastrointestinal inflammation and AD (Pessi et al., 2000). Kirjavainen et al. (2003) reported lower *Bacteroides* counts in the fecal microflora of children with atopic eczema than in healthy infants and suggested that probiotics can be used to treat AD in children (Kirjavainen et al., 2003); however, some reports yielded contrasting results (Licari et al., 2015). For instance, Gruber et al. found that *Lactobacillus rhamnosus strain GG (LGG)* exerted no therapeutic effects in infants with mild-to-moderate AD (Gruber et al., 2007). Therefore, we systematically evaluated the effects of probiotics used to treat AD in children.

## Methods

### Inclusion criteria

The inclusion criteria for the meta-analysis were (1) RCTs of children aged ≤ 18 years in whom AD severity was graded by experienced dermatologists using the Severity scoring of atopic dermatitis: the SCORAD index (1993); Yoon et al. (2015) (2) that evaluated the use of any probiotic culture/strain/dose/therapy regimen (including studies on fermented yogurt; all dosage forms including tablets, powders, oil suspensions, and capsules were included). All results are presented as means ± standard deviation. However, if multiple reports evaluated the same group of patients, we selected only the most recent complete report. SCORAD, developed by the European Task Force on AD in 1993 (1993), assesses the AD area, clinical features, visual analog scale data, and clinical symptoms, and it is widely used to evaluate AD severity in children (Machura et al., 2008).

### Exclusion criteria

Studies that did not meet the inclusion criteria or that were published in languages other than English were excluded.

### Search process

Two individuals of our team searched the following databases from the times of the earliest records in 2000 to April 12, 2017: PubMed (https://www.ncbi.nlm.nih.gov/pubmed), Embase (https://www.embase.com/login), Cochrane Library (http://www.cochranelibrary.com/) and Scopus (https://www.elsevier.com/solutions/scopus) (available on the internet); and Ovid, Orbis, and the Web of Science (available at our university library with free downloads). The following search string was used in searching: [(infant OR infants) OR (neonate OR neonates) OR (newborn OR newborns) OR (toddler OR toddlers)] AND (probiotic OR probiotics OR pro-biotics OR probio^*^) AND (atopic dermatitis OR atopic eczema) OR (SCORAD) OR (atopic OR atopy) NOT (animals) NOT (adult). The references listed in each report were examined to allow us to retrieve additional information. We only reviewed works in the English language, thus not those in (for example) Korean or Chinese. Furthermore, conference abstracts were excluded, because they lacked detailed data.

### Data collection

The two individuals collected all data independently. The eligibility of studies was confirmed by both reviewers. A tabulation of study author(s), publication date, recruited numbers, probiotic strain(s), dosage, treatment duration, and treatment results was prepared (Table 1). If the study data were unclear, we attempted to contact the corresponding author via email to obtain further information.

**Table 1:** Characteristics of included RCTs for meta-analysis.

**Study, year (country)**	**n**	**Age**	**Genus, species, and strain, duration**	**Dose**	**Outcome summary**
Viljanen et al., 2005; Finland	220	1.4–11.9 months	*Lactobacillus rhamnosus strain GG (LGG)*; 4 weeks	5 × 10^9^ cfu or mixture twice daily capsules	Positive effect of probiotics was seen only in IgE-sensitized infants
Weston et al., 2005; Austria	56	6–18 months	*LF; 8-week*	2 × 10^10^ CFU/g/d	Positive effect of probiotics was seen only in food-sensitized children
Folster-Holst et al., 2006; Germany	54	1–55 months	*Lactobacillus rhamnosus strain GG(LGG); 8-week*	10 × 10^9^CFU	No significant difference between synbiotics and placebo
Gruber et al., 2007; Germany	102	3–12 months	*Lactobacillus rhamnosus strain GG(LGG); 8-week*	>5 × 10^9^CFU, twice daily orally	No significant difference between synbiotics and placebo
Niers et al., 2009; Netherland	98	1–24 months	*B. bifidobacterium infantis, LC. lactis W58; 24-week*	3 × 10^9^CFU, once daily	No difference was observed among two groups
Wu et al., 2012; Taiwan	60	2–14 years	*Lactobacillus (LS), 8-week*	5 × 10^10^ CFU, twice daily	SCORAD decrease significantly in probiotic group compared to placebo group
Gerasimov et al., 2010; Ukraine	90	1–3 years	*Mixture (LA t BL)/synbiotics; 8-week*	5 × 10^10^ CFU, twice daily	SCORAD decrease significantly in probiotic group compared to placebo group
Woo et al., 2010; Korea	75	2–10 years	*Lactobacillus (LS2)/synbiotics; 12-week*	2 × 10^10^ CFU, twice daily	SCORAD decrease significantly in probiotic group compared to placebo group
Shafiei et al., 2011; Iran	41	1–36 months	*Seven strain probiotics plus prebiotic mixture; 2 months*	1 × 10^9^CFU, once daily	No significant difference between probiotics and placebo
Gore et al., 2012; UK	133	3–6 months	*Lactobacillus (LP) or Bifidobacterium (BL); 12-week*	1 × 10^10^ CFU	No significant difference between probiotics and placebo
Han et al., 2012; Korea	83	1–13 years	*Lactobacillus (LP2); 12-week*	5 × 10^10^ CFU, twice daily	SCORAD decrease significantly in probiotic group compared to placebo
Yesilova et al., 2012; Turkey	39	1–12 years	*Mixture (BB2, LA, LC, LS2); 8-week*	4 × 10^10^ CFU, daily	SCORAD decrease significantly in probiotic group compared to placebo
Wang and Wang, 2015; Taiwan	220	1–28 years	*Lactobacillus paracasei(LP), Lactobacillus fermentum(LF), Mixture; 3 months*	LP, LF(2 × 10^10^ CFU, qd); Mixture(4 × 10^10^ CFU, qd)	SCORAD decrease significantly in probiotic group compared to placebo

### Statistical analysis

RevMan 5.3 software (Cochrane Collaboration, Nordic Cochrane Center, Copenhagen, Denmark; http://community.cochrane.org/tools/review-production-tools/revman-5/) was accessed to conduct the meta-analysis. SCORAD was commonly used to measure the efficacy of probiotics in children with AD. As the results were continuous data, the mean difference (MD) and 95%CI were calculated for statistical analyses, and either a randomized-effects model or fixed-effects model was used depending on whether heterogeneity was apparent. Subgroup assessment was performed with regard to different geographical status, infants aged < 1 year, children aged between 1 and 18 years, different strains, and *LGG*. The c^2^ test was used to identify statistical heterogeneity (Margolis and Mitra, 2017). The I^2^ statistic was calculated to identify and quantify inconsistency. When I^2^ was ≥ 50%, indicating significant heterogeneity, we used a random-effects model for meta-analysis. When I^2^ was < 50% indicating no heterogeneity, we employed a fixed-effects model. Publication bias was assessed by constructing funnel plots. A two-tailed *P* < 0.05 was used to reflect statistical significance. Sensitivity analyses, also termed uncertainty analyses, were used to explore the extent to which our results and conclusions were altered by changes in the data or analysis approach (Alexander et al., 2016). If the conclusions did not change upon application of the sensitivity analysis, those conclusions were considered robust. In meta-analyses, sensitivity analyses are conducted by excluding studies one-by-one to identify those studies that materially affect the results (Copas and Shi, 2000). The risk of bias in each RCT was explored using the “risk of risk” tool in Revman software. The PRISMA statement published in 2009 aimed to improve the reporting of systematic reviews and meta-analyses. PRISMA defines an evidence-based minimum set of items to employ, and we followed this guideline (http://www.prisma-statement.org/). PRISMA features both a checklist and a flow diagram. We used the checklist to ensure that our study structure was appropriate and the flow diagram to map the numbers of records identified, included, and excluded, as well as the reasons for exclusion (Zhang et al., 2017). Publication bias was checked by drawing funnel plots, which are commonly used in systematic reviews and meta-analyses. Publication bias is considered absent if the study results are distributed in close proximity to the averages.

## Results

### Included studies

**Figure 1:**
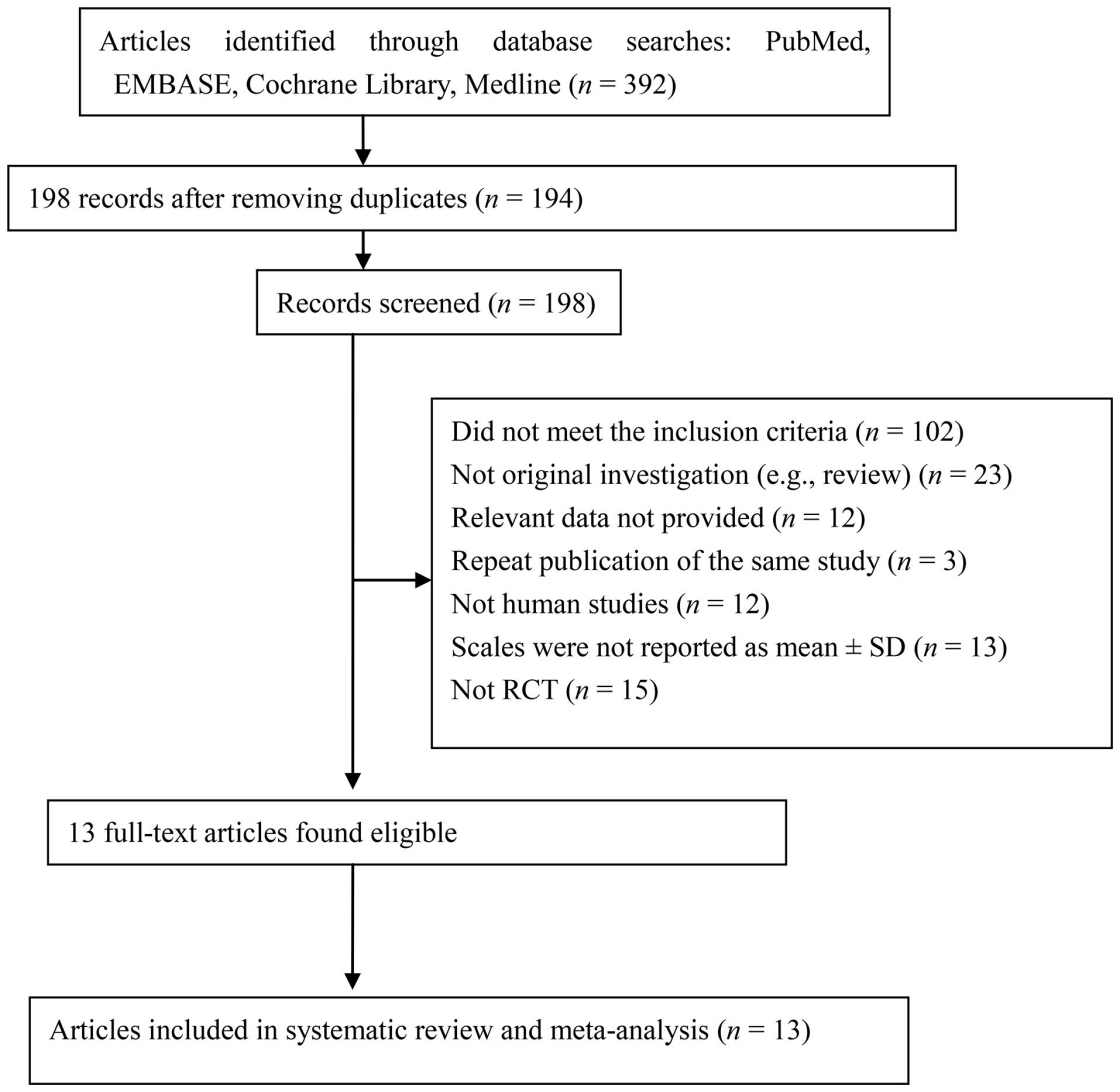
PRISMA flow diagram of articles included in the meta-analysis.

The PRISMA flow diagram (Figure 1) shows how we selected the relevant reports. We initially screened 392 articles, excluded those that did not meet our inclusion criteria, and finally retained 26 articles. As some reports did not report data as means ± SD, we contacted the corresponding authors by email. Unfortunately, we sent 13 emails and didn't receive any data suitable for inclusion in the meta-analysis. Ultimately, 13 studies involving 1,070 children fulfilled our selection criteria (Table 1).

### Quality assessment

**Figure 2:**
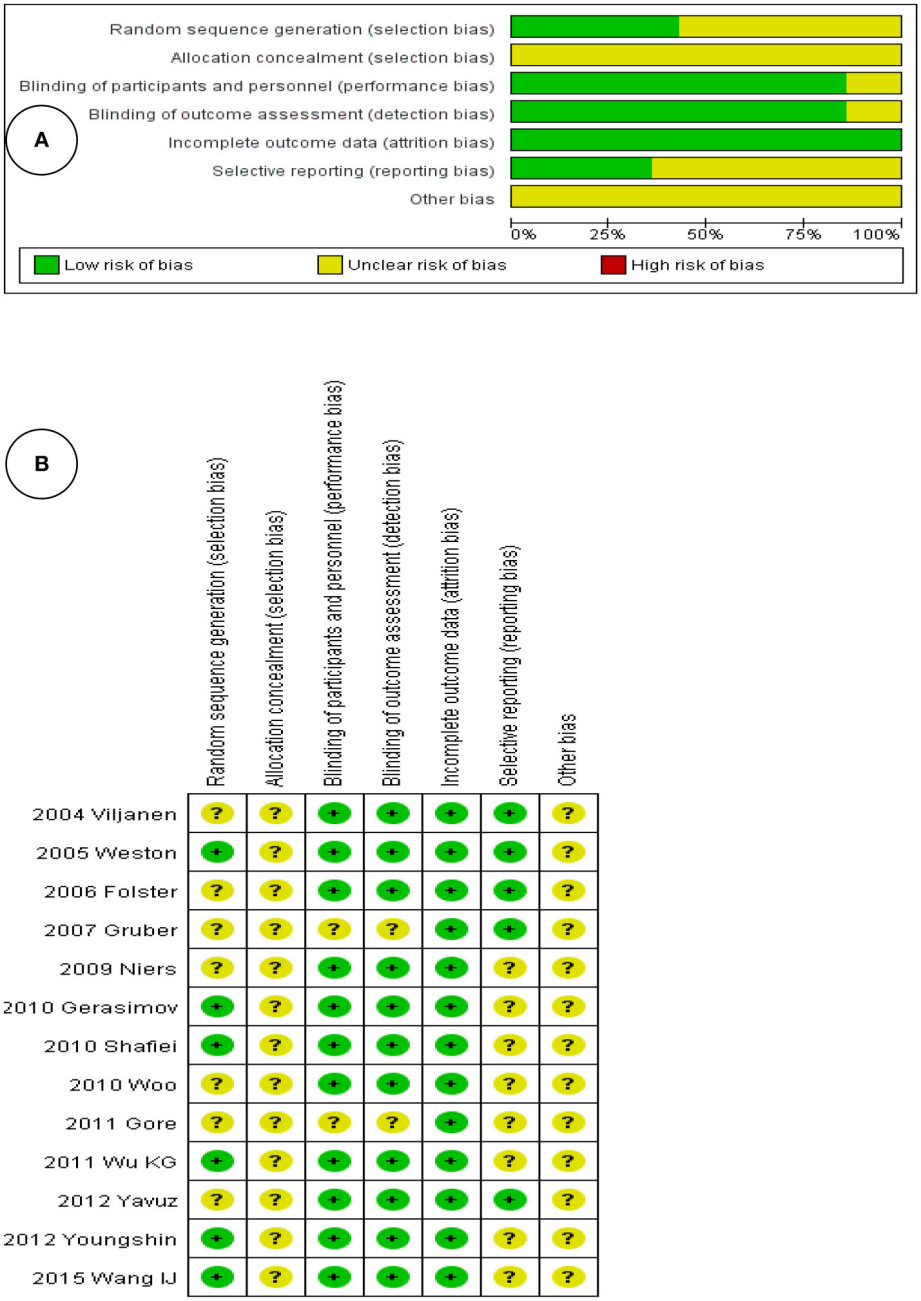
**(A)** Risk of bias graph, with each risk of bias item presented as a percentage across all included studies. **(B)** Risk of bias summary, with each risk of bias item for each included study.

Figure 2A shows the risk of bias within all enrolled RCTs, as adjudged by the two reviewers. Figure 2B presents the individual risks of bias, again as perceived by the reviewers. Both figures show that the risks of bias were rather low, because all were RCTs that adhered to high standards. Four studies divided children into probiotic intervention and control groups; two studies created three groups (probiotics, a placebo, and another intervention). Twelve studies were of double-blind design. All 13 studies reported baseline data including socioeconomic status and mean age; these did not differ significantly among the groups.

### Probiotics and children with AD

**Figure 3:**
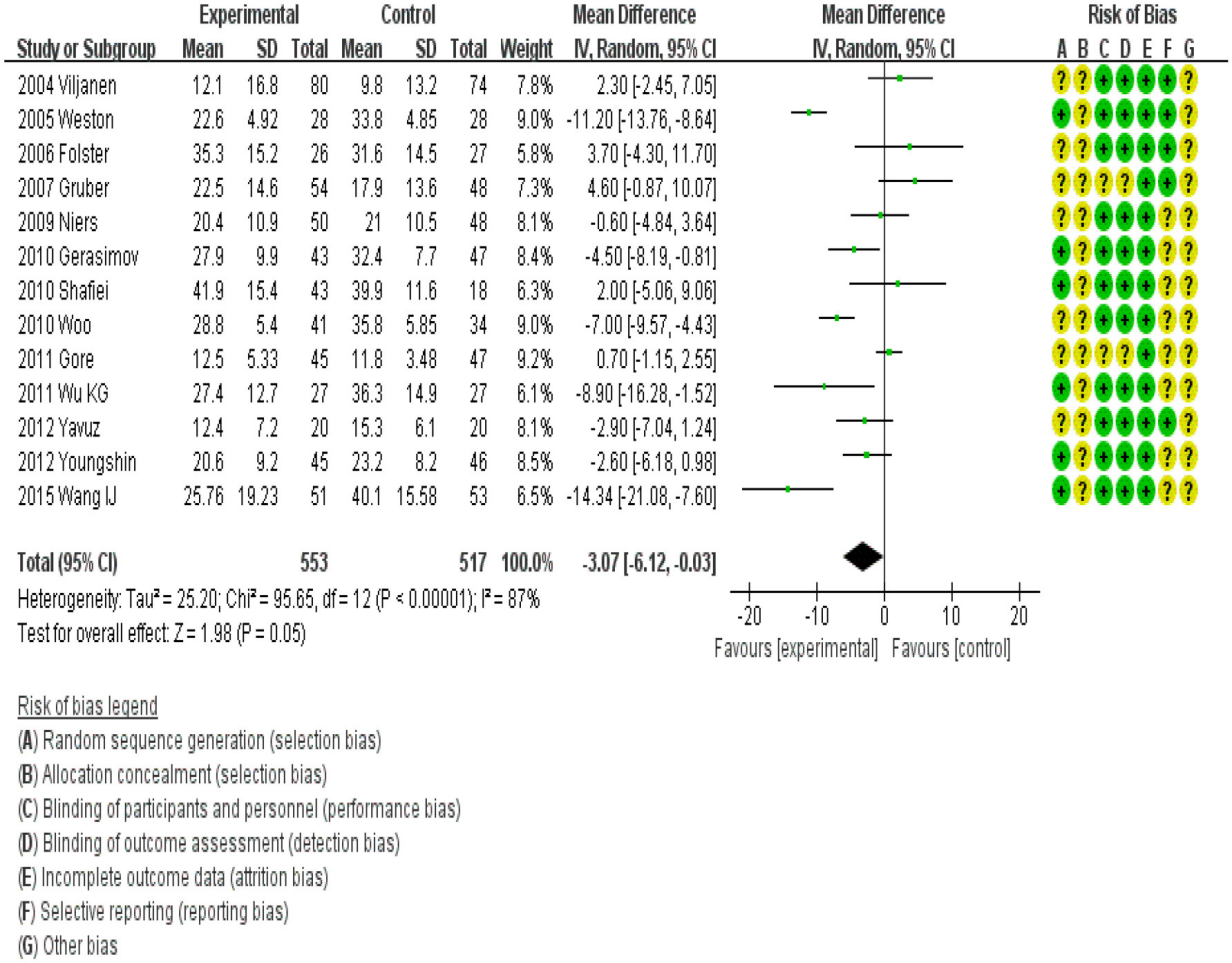
MD scoring with probiotics treatment compared to control and placebo interventions. 95%CI, 95% confidence interval.

Data from 1,070 children (intervention group, 553; control group, 517) were assessed. The outcome of a random-effects meta-analysis model involving all 13 trials is shown in Figure 3. Significant differences in SCORAD values favoring probiotics over the control were observed overall (MD, −3.07; 95%CI, −6.12 to −0.03; *P* < 0.00001). However, a high degree of heterogeneity was observed across these 14 trials (I^2^ = 87%).

### Subgroup analysis of probiotics efficacy by age

**Figure 4:**
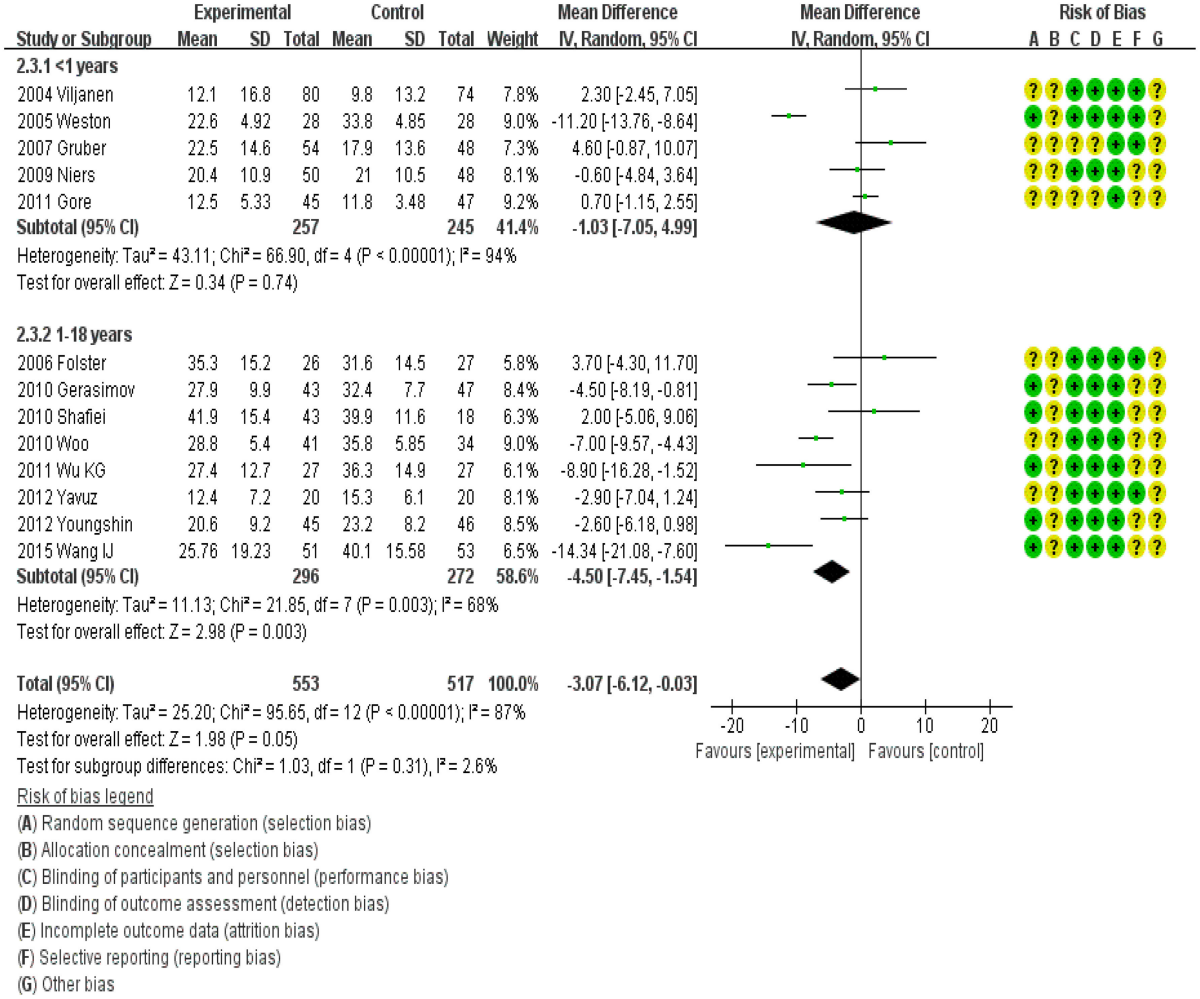
MD scoring with probiotics treatment compared to control and placebo interventions by age group.

All 13 trials involved children aged 0–18 years. We categorized the children into two groups: infants < 1 year old and children 1–18 years old. Accordingly, five trials were included in the < 1 year subgroup, and nine trials were included in the 1–18 years subgroup (Figure 4). The efficacy of probiotics in the former subgroup was −1.03 (95%CI, −7.05 to 4.99) and that in the latter subgroup was −4.50 (95%CI, −7.45 to −1.54; *P* < 0.001). However, a high degree of heterogeneity was observed among the < 1 year subgroup (I^2^ = 94%).

### Subgroup assessment by continent

**Figure 5:**
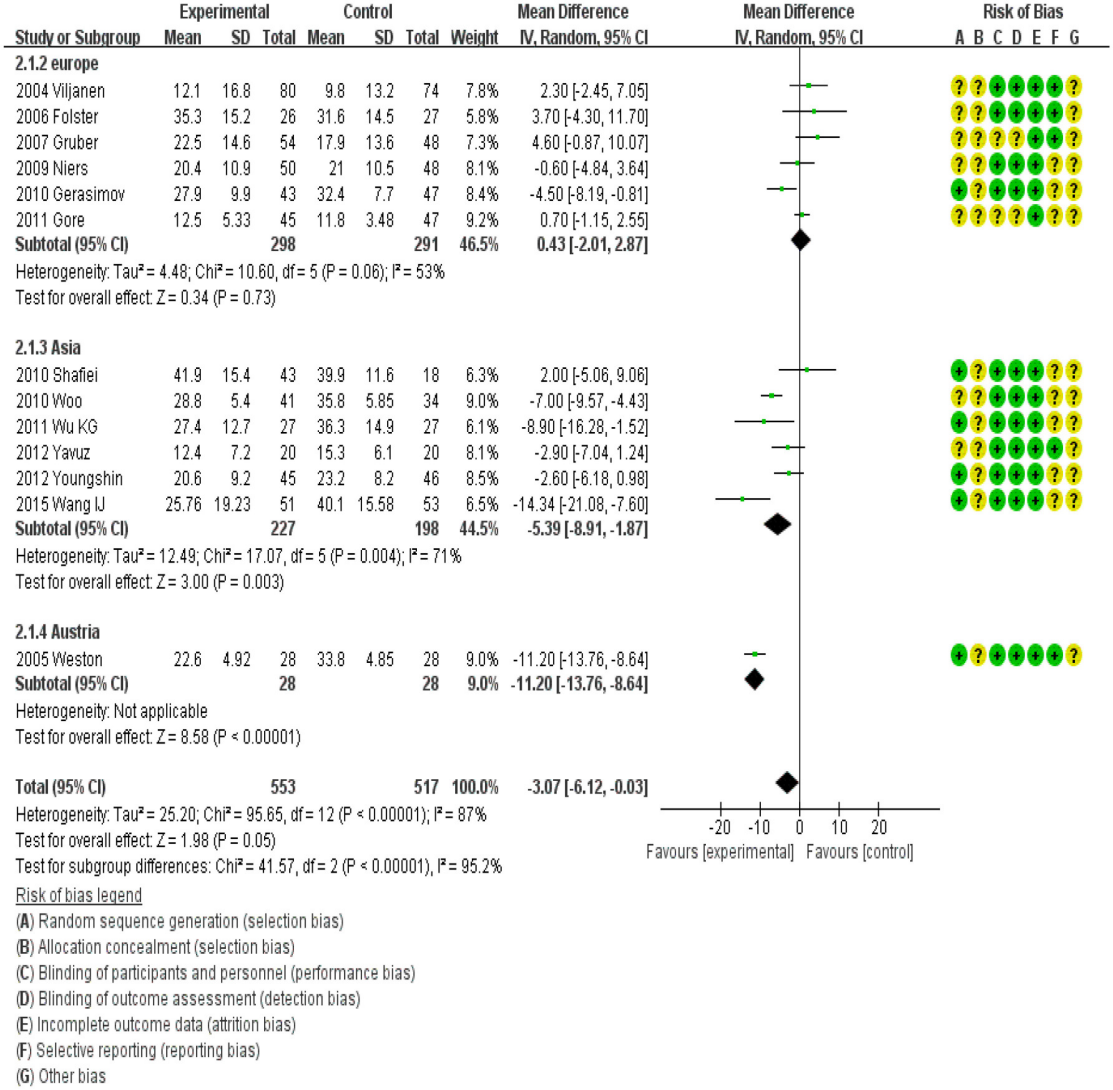
MD scoring with probiotics treatment compared to control and placebo interventions by location.

Subgroup assessment by continent showed different effects. In Europe, probiotics showed no effect on SCORAD, whereas significantly lower SCORAD values were reported in Asia (MD, −5.39; 95%CI, −8.91 to −1.87). In Australia, the MD was −11.20 (95%CI, −13.76 to −8.64). However, there was heterogeneity among these trials (Figure 5).

### Subgroup assessment of different cultured organisms

**Figure 6:**
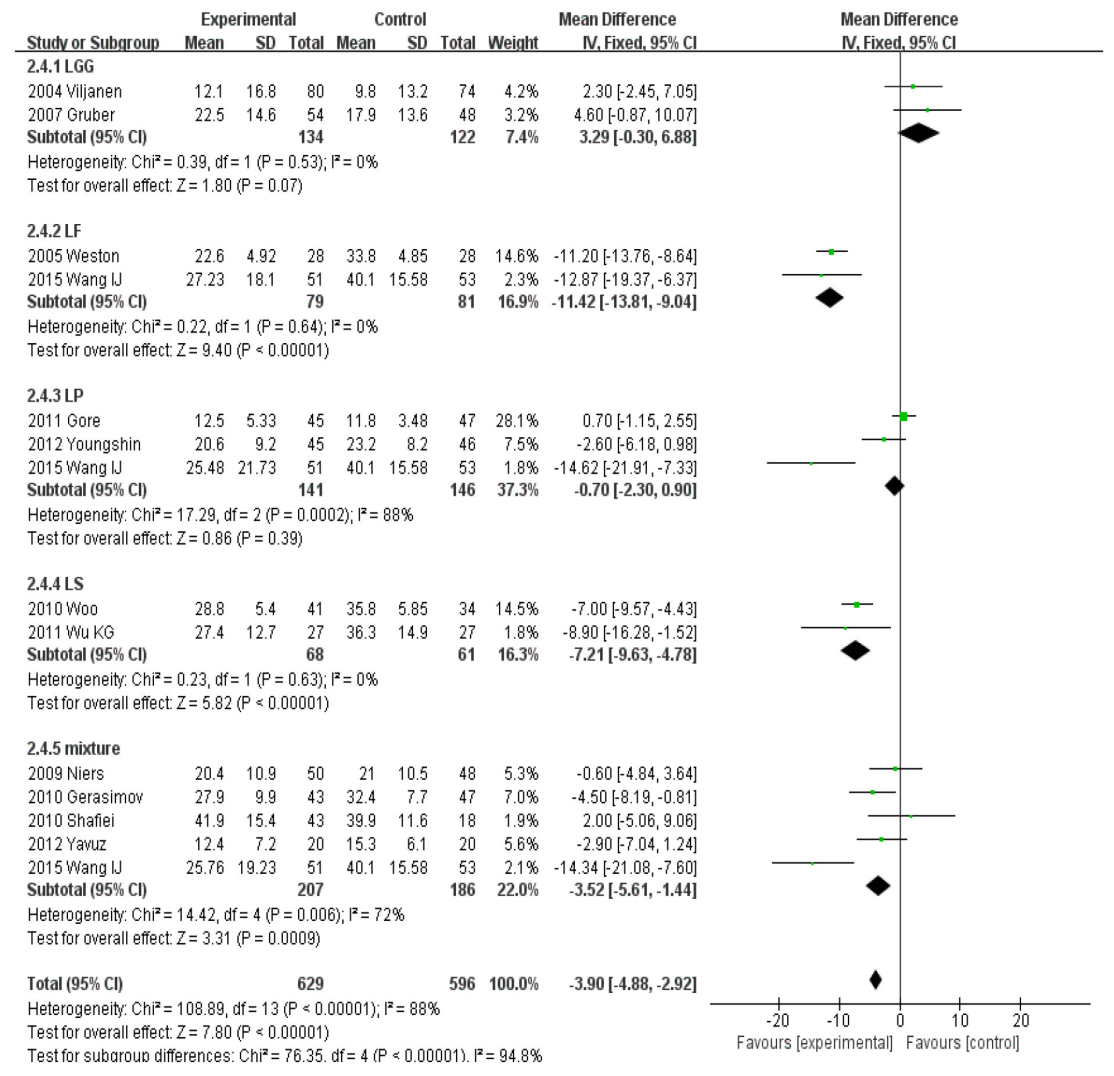
MD scoring with probiotics treatment compared to control and placebo interventions by cultured organisms.

MD scoring compared to control and placebo interventions was performed by cultured organism group. *LGG* (MD, 3.29; 95%CI, −0.30 to 6.88; *P* = 0.07) and *LP* (MD, −0.70; 95%CI, −2.30 to 0.90; *P* = 0.39) showed no significant effects on SCORAD values in children. However, *LF* (MD, −11.42; 95%CI, −13.81 to −9.04), *LS* (MD, −7.21; 95%CI, −9.63 to −4.78), and a mixture of different strains (MD, −3.52; 95%CI, −5.61 to −1.44) showed significant effects on SCORAD values in children (Figure 6).

### Publication bias

**Figure 7:**
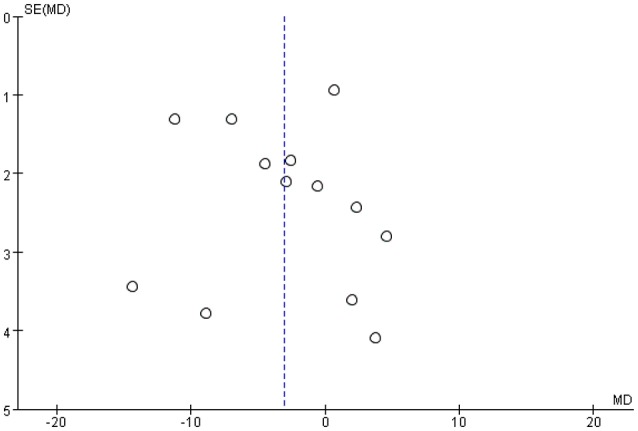
Funnel plot: SD by mean difference and each dot represents 1 RCT.

We used RevMan software to draw funnel plots (Figure 7), wherein each dot represents data from a single RCT. A random-effects model was used to this end. The funnel plots were somewhat asymmetrical, thus indicating potential publication bias, perhaps attributable in part to the fact that we included only English-language publications and excluded conference abstracts. However, studies with positive outcomes are more likely to be published than are those with negative outcomes, thus creating bias.

### Sensitivity testing

We performed sensitivity analyses to assess the relative influence of each study by excluding the studies one by one, and the results suggested no significant changes in effects with regard to subgroups.

## Discussion

Overall, the data suggested an overall benefit of probiotics supplementation in children with AD, and age-specific sub-analyses showed that probiotics effectively reduce SCORAD values in children aged 1–18 years. Geography-specific sub-analyses showed that probiotics effectively reduced SCORAD values in Asia, while no effect was observed for Europe. Strain-specific sub-analyses indicated that *Lactobacillus (LS), Lactobacillus fermentum (LF)*, and a probiotic mixture reduced SCORAD values in children with AD, while LGG and Lactobacillus plantarum (LP) showed no effect in children with AD.

Hippocrates (460–370) stated that “All diseases begin in the gut”, which is the earliest suggestion that bacteria affect health (Hippocrates, 2002). Metchnikoff, known as the father of probiotics (Gordon, 2016), proposed that colonic bacteria afforded health benefits in aging adults. In recent decades, probiotics that aid in the resolution of pediatric atopic eczema have been investigated. Viljanen et al. explored probiotic effects on pediatric atopic eczema/dermatitis syndrome but found no significant difference between the treatment and control groups (Viljanen et al., 2005). Passeron et al. compared probiotics and prebiotics and found that both significantly improved AD manifestations in children (Passeron et al., 2006). Brouwer et al. evaluated the clinical and immunological effects of *Lactobacillus rhamnosus* (*LR*) supplementation in a hydrolyzed formula given to children with AD but found no significant effect (Brouwer et al., 2006). The cited authors suggested that the discrepancies between their results and those of other trials were likely attributable to differences in treatment timing and the strains used. Sistek et al. conducted a 12-week trial in the UK and found that a combination of *LR* and *Bifidobacteria lactis* (*BL*) improved AD symptoms in food-sensitive children (Sistek et al., 2006). At roughly the same time, a prospective German study by Folster-Holst et al. yielded insufficient evidence to make the conclusion that *LGG* is an effective treatment for moderate-to-severe AD in infants (Folster-Holst, 2010). Gruber et al. also found that *LGG* had no therapeutic effect in such patients (Gruber et al., 2007). Despite these discouraging findings, Gerasimov et al. reported that *Lactobacillus acidophilus DDS-1* and *Bifidobacterium lactis UABLA-12* afforded significant clinical improvements in children with moderate-to-severe AD (Gerasimov et al., 2010). Similarly, Wu et al. showed that *Lactobacillus salivarius* (*LS*) exerted short-term beneficial effects in patients with moderate-to-severe AD (Wu et al., 2012). Drago et al. suggested that such effects may be attributable to restoration of the altered intestinal microbiota (Drago et al., 2011). In contrast, Gore et al. found that *LS* exerted no beneficial effects on eczema when given as an adjunct to basic topical treatment (Gore et al., 2012). Several reports have examined the effects of other bacterial strains on AD in children. Supplementation with *LPCJLP 133, Lactobacillus paracasei*, and *LF* was reported to be effective. The discrepancies described above may be attributable to differences in the strains used, the study areas, and/or the ethnicities of the subjects. Several groups have performed meta-analyses to evaluate the effectiveness of probiotics on AD. Da Costa Baptista et al. reviewed all published trials and reported that the biological effects observed in most trials suggest that probiotic adjuvant treatments are of benefit for AD (da et al., 2013). The cited review, although comprehensive, did not report total MDs or 95%CIs. Chang performed a meta-analysis of studies in which either prebiotics or probiotics were given and reported that synbiotics may be useful to treat AD (Chang et al., 2017). However, the focus was on synbiotics rather than probiotics. Szajewska et al. stressed the need for data on individual probiotic strains rather than on probiotics in general (Szajewska and Mrukowicz, 2003; Szajewska et al., 2015). Ogden et al. suggested probiotics as a complementary approach to the treatment and prevention of pediatric AD (Ogden and Bielory, 2005). They concluded that probiotics should be an active area of investigation, considering the role of gut microbiota in altered immune responses in atopic patients. However, the authors did not perform a meta-analysis to obtain further details about the treatment effects of probiotics. Kim et al. reviewed 25 RCTs on the effects of probiotics in the treatment of AD in patients of all ages. They observed significant differences in SCORAD values favoring probiotics over the control group in children 1–18 years old and in adults, whereas no favorable effects were seen in infants < 1 year old (Kim et al., 2014). We found that probiotics were efficacious in children aged 1–18 years (MD, −4.50; 95%CI, −7.45 to −1.54) and showed strong efficacy in Asia but not in Europe; furthermore, *LGG* had no effects on AD whereas *LS, LF, LP*, and a mixture of strains showed beneficial effects. Our findings are in agreement with those of Lee et al., who concluded that the evidence for probiotics as a useful treatment of AD in children is convincing. However, the cited authors reviewed only trials published before 2008, whereas we included later trials to afford greater insight. The differences may be because we included only RCTs involving children under the age of 18 years and those that reported MDs. Some RCTs presented values other than MDs, including the study by Kim et al. (2010), and some presented the results as figures, rendering calculations impossible. We contacted the corresponding authors but did not receive useful replies. Thus, we excluded those studies. Third, some of the included studies had small sample sizes, which may affect the reliability and validity of the conclusions. Thus, our overall results are affected by these issues, and the data were highly heterogeneous. These topics require further attention. Also, in the subgroup analyses, children with AD may have different gut microbiota profiles from those of normal children. Thus, probiotics supplementation in children < 1 year old and 1–18 years old may promote a healthier gut microbiota profile, boosting their immune response. People from different areas have different dietary structures and gut bacterial compositions. Dehingia et al. compared gut bacterial diversity between Indian populations and worldwide data (Dehingia et al., 2015). Zhang et al. also suggested that a phylogenetically diverse gut microbiota at the genus level may be commonly shared by distinctive healthy populations, which may explain the diversity of the effects of probiotics across people from different countries (Zhang et al., 2015). The above discussion is of importance to physicians, dermatologists, and other public healthcare workers who deal with diverse ethnic populations.

To the best of our knowledge, there are no previous reports on the effects of different probiotic strains on AD in children. In our meta-analysis, all trials involving *LGG* and one trial involving *LP* showed no effects, while two studies confirmed the beneficial effects of *LP* on AD (MD, −0.70; 95%CI, −2.30 to 0.90; *P* = 0.39). This discrepancy may be associated with differences in dosages, the timing and duration of intervention, and sample sizes, and further trials are required to clarify this point.

Our meta-analysis had certain limitations. First, we attempted to minimize heterogeneity and publication bias, but significant heterogeneity among trials remained evident. Differences in study samples, study populations, and intervention methods contributed to the heterogeneity. For example, some of the included studies had small sample sizes, compromising the reliability and validity of the conclusions. In addition, the RCTs were performed in various countries, thus, the subjects differed among RCTs in terms of their genetic make-up and microbial exposure, which in turn are associated with varying responses to the same probiotic. Also, we excluded some RCTs from this meta-analysis, and fewer studies included will reduce the confidence associated with the data interpretation and increase heterogeneity and publication bias. Finally, we cannot draw robust conclusions as to which probiotic strain/mixture should be given to children with AD and which population(s) would receive maximum benefit from such treatment.

## Conclusion

Our present work demonstrated that probiotics may have the potential to decrease SCORAD values in children with AD. However, the findings presented here must be generalized with caution because of heterogeneity. The results are a source of optimism with regard to the management of AD in children. More adequately powered RCTs using standardized measurements are necessary to assess which species of probiotics and dosages and what treatment periods are most efficacious for children with AD.

## Author contributions

RH, MS, and XC proposed the idea of this study and designed the study; RH, HN, MS, and JL conducted data screening and performed quality assessment; RH and MS used RevMan software to assess the data and performed the statistical analysis and gave the explanations of the statistical results. RH drafted the initial manuscript. JL, JZ, and XC critically reviewed and revised the manuscript.

### Conflict of interest statement

The authors declare that the research was conducted in the absence of any commercial or financial relationships that could be construed as a potential conflict of interest.
